# Dangers of Noncritical Use of Historical Plague Data

**DOI:** 10.3201/eid2401.170477

**Published:** 2018-01

**Authors:** Joris Roosen, Daniel R. Curtis

**Affiliations:** Utrecht University, Utrecht, the Netherlands (J. Roosen);; Leiden University, Leiden, the Netherlands (D.R. Curtis)

**Keywords:** plague, Black Death, data, history, databases, mapping, sources, epidemics, bacteria, vector-borne infections, zoonoses, high-consequence pathogens

## Abstract

Researchers have published several articles using historical data sets on plague epidemics using impressive digital databases that contain thousands of recorded outbreaks across Europe over the past several centuries. Through the digitization of preexisting data sets, scholars have unprecedented access to the historical record of plague occurrences. However, although these databases offer new research opportunities, noncritical use and reproduction of preexisting data sets can also limit our understanding of how infectious diseases evolved. Many scholars have performed investigations using Jean-Noël Biraben’s data, which contains information on mentions of plague from various kinds of sources, many of which were not cited. When scholars fail to apply source criticism or do not reflect on the content of the data they use, the reliability of their results becomes highly questionable. Researchers using these databases going forward need to verify and restrict content spatially and temporally, and historians should be encouraged to compile the work.

In an article by Jones and Nevell (*1*), the authors argue that improved access to historical data through digitization projects has benefited research in different scientific fields. However, they also point out that digitization has some unintended consequences. A key issue they identified is the loosening of the rigorous standards of evidence and interpretation scientific researchers typically demand within their own disciplines (*1*). Although scholars regularly reprimand colleagues for misrepresenting evidence and misusing data to make arguments that their material cannot support, such issues are less frequently addressed when data sets transcend the border from one scientific discipline to the next. This discrepancy poses a problem in an age of greater interdisciplinary research.

Here we focus on the most frequently used record of historical plague outbreaks in Europe. This information was originally compiled >40 years ago by Jean-Noël Biraben as part of his 2-volume work, Les hommes et la peste en France et dans les pays méditerranéens, which documents plague outbreaks from the Black Death (1347–1352) to the 19th century (*2*,*3*). Using a digitized version of this data set (https://zenodo.org/record/14973), which includes a limited number of outbreaks in northern Africa, authors have boasted impressive collections of documented European plague outbreaks: 6,929 plague outbreaks across Europe during 1347–1900 (*4*), 7,711 outbreaks across Europe and Asia during 1347–1900 (*5*), 5,559 outbreaks across Europe and northern Africa during 1347–1760 (*6*), and 6,656 outbreaks across Europe during 1347–1760 (*7*). In one of these studies, the Biraben data set was supplemented with additional outbreaks from Russia and Turkey gleaned from secondary literature (*5*).

Biraben had the ambition of constructing a pan-European overview of recurring plague outbreaks, and although his work at the time was an extraordinary feat of scholarship, a complete documentation of the occurrence of plague throughout Europe could not be adequately concluded by any single researcher. From a historian’s perspective, the most fundamental problem with Biraben’s data is the lack of systematic justification for the sources used and only cursory referencing of the original documents. However, this article is not meant to be a criticism of Biraben’s 1970s work but of the research published decades later by authors who interpreted Biraben’s results at face value. Scholars who have used this data set have not applied adequate source critique expected within the field of history, failing to pose basic questions concerning how the data were collected and what they represent. The 4 aforementioned studies (*4*–*7*) are not the only instances in which the Biraben data set were not used critically; in fact, there are many examples (*8*–*13*). However, Büntgen et al., Schmid et al., and Yue et al. are the first to use a digitized version of the data set, which not only causes specific problems but also sets a dangerous precedent for future research (*4*–*7*).

## Noncritical Use of Historical Plague Databases

In 2012, Büntgen et al. presented the digitized version of the Biraben data set in a short correspondence piece (*4*). This publication reflected little on the limitations of the data. The only concerns Büntgen et al. addressed were the imprecise geographic descriptions that impeded exact localization and the annual resolution of the data that precluded tracking of outbreaks within the same year. However, through digitization and subsequent publication in a top-ranked journal, the 4-decade-old data set was imbued with a false aura of trustworthiness and the impression of being new historical research. Subsequently, others used the resulting database noncritically, in some cases not referencing the original Biraben data at all (*6*). The perpetual reuse of these data without structural effort to add new archival evidence has given the impression that our knowledge of historical plague outbreaks is saturated and, moreover, has obscured the fact that large amounts of innovative research on the spatiotemporal spread of plague has been conducted by others since the mid-1970s.

**Figure 1:**
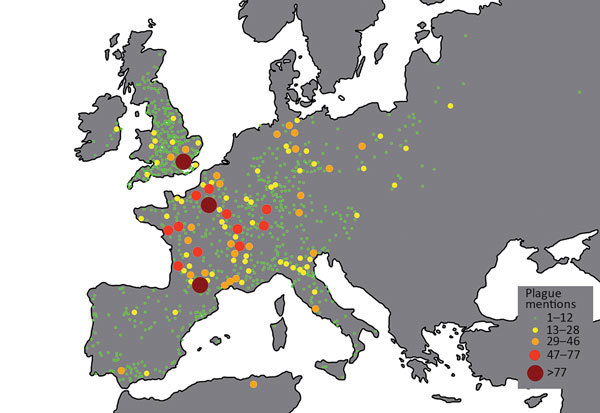
Plague outbreaks in Europe, 1347–1760. Map produced on the basis of data from Biraben (*2**,**3*). Map provided courtesy of Yue et al. Navigable rivers facilitated the spread and recurrence of plague in pre-industrial Europe. Sci Rep. 2016;6:34867 (*6*).

These problems can be demonstrated through the maps that have been produced on the basis of Biraben’s data. Büntgen et al. provided a map in the introduction to the database (*4*), and a copy (Figure 1) was later included in an article on the supposed link between plague spread and navigable rivers (*6*). However, looking at the map, 2 problems surface immediately. First, France is depicted as the major epicenter of plague activity across 4 centuries, something even accepted as a face-value truth by some scholars (*13*). However, more than likely, the concentration of plague activity reflected nothing more than the fact that Biraben was French and had exceptional knowledge of the archives in France (*14*). Second, there are vast areas where no plague was recorded across the whole of the late-medieval and early modern periods. For example, hardly any recorded plague outbreaks appear in a period of 4 centuries in much of the Low Countries in western Europe.

**Figure 2:**
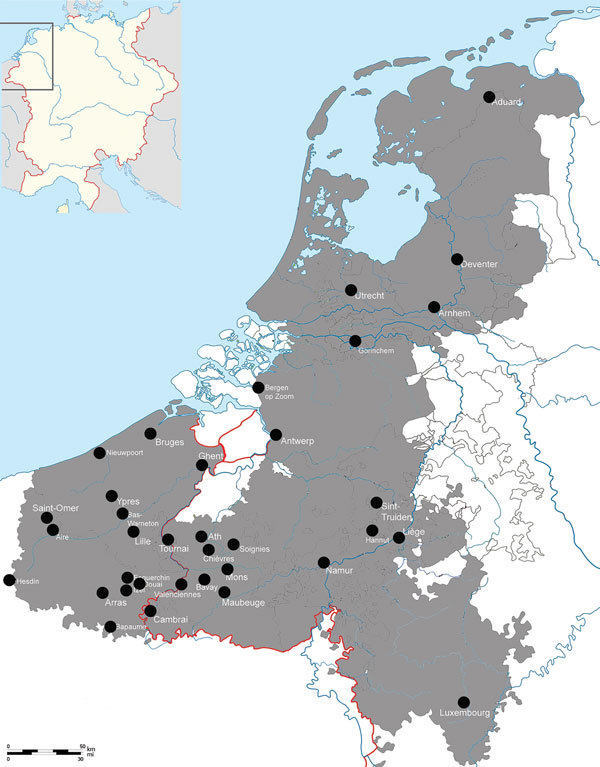
Plague mentions during the Black Death outbreak, Low Countries, 1348–1352 (*18*). Inset shows location of the Low Countries in western Europe.

**Figure 3:**
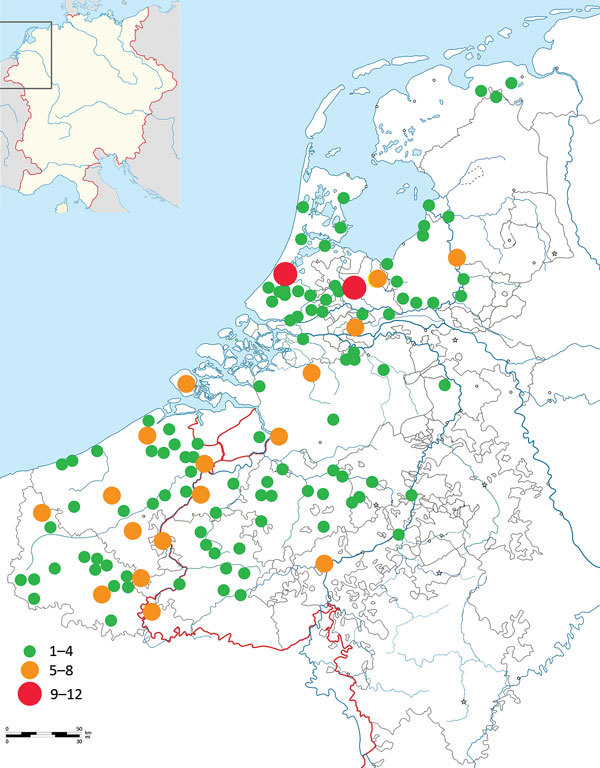
Plague mentions taken from archival sources, Low Countries, 1348–1500 (*18*). Inset shows location of the Low Countries in western Europe.

If we were to focus exclusively on the initial Black Death outbreak (1347–1352), this evidence would be in agreement with the literature of the mid-1970s. At that time, the consensus was that the Black Death somehow did not reach most parts of the Low Countries (*2*,*15*,*16*). Later this view was refuted, and proof that the Black Death was present in the Low Countries was established (*17*). In fact, a newly compiled data set of plague mentions shows that many regions of the Low Countries were hit by the Black Death (Figure 2) (*18*). When we add data of plague mentions across the entirety of the late Middle Ages (1349–1500), this map becomes filled to an even greater extent (Figure 3) (*18*), without even adding plague data from the 16th and 17th centuries, when many plagues, such as those in 1624‒1625 and 1635‒1636, hit almost every recordable locality of the Low Countries, both urban and rural (*19*). Biraben’s data set, therefore, is not only hindered by being outdated but also by having crucial gaps in spatial coverage, leaving out large parts of the Low Countries, Denmark, Scotland, Ireland, and Central Europe (*20*). Even countries well known in the literature for having experienced numerous plagues of exceptional severity across the Middle Ages and during the early modern period, such as Italy (*14*,*21*), have very few plague markers on the maps produced with Biraben’s data.

## Biraben Data Set

In the examples we mention, 3 transgressions have been attributed to the scholars using the Biraben data set. First, reflection on the data collection process has been improper; second, what the data represent has not been recognized; and third, critique of the original sources has been inadequate. We argued that a critical consideration of any of these 3 elements would have led to the conclusion that the data set could not have been used at face value.

**Figure 4:**
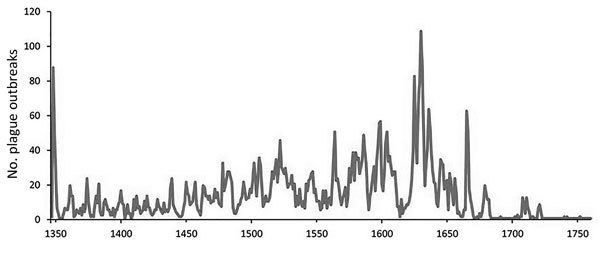
Plague outbreaks in Europe, 1347–1900. Graph produced on the basis of data from Biraben (*2**,**3*). Graph provided courtesy of Büntgen U et al. Digitizing historical plague. Clin Infect Dis. 2012;55(11):1586‒8 (*4*). By permission of Oxford University Press.

**Figure 5:**
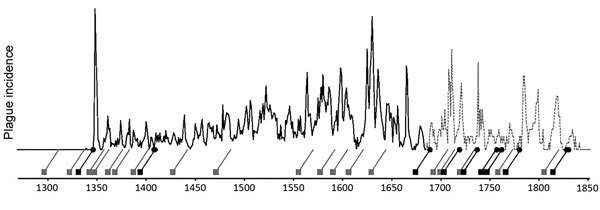
Plague incidences in Europe, 1347–1900. Graph produced on the basis of data from Biraben (*2**,**3*). Graph provided courtesy of Schmid BV et al. Climate-driven introduction of the Black Death and successive plague reintroductions into Europe. Proc Natl Acad Sci U S A. 2015;112:3020‒5 (*5*).

**Figure 6:**
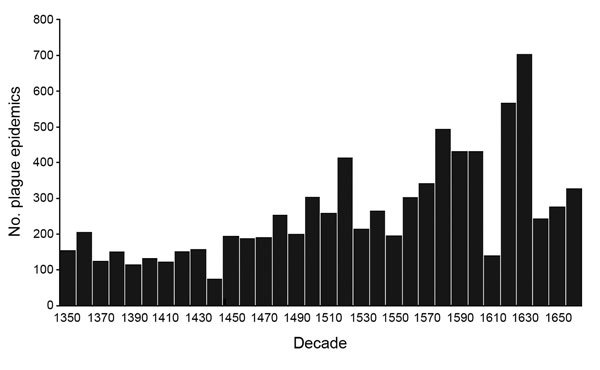
Plague epidemics in Europe, 1350s–1660s. Graph produced on the basis of data from Biraben (*2**,**3*). Modified graph provided courtesy of Voigtländer N, Voth H-J. Gifts of Mars: warfare and Europe’s rise to riches. J Econ Perspect. 2013;27:165‒86 (*12*).

First, we address what the Biraben data represent. Three previously published graphs display the same data set (Figures 4,5,6) and yet, peculiarly, present the data differently. Büntgen et al. described the data as plague outbreaks, Schmid et al. referred to the data as plague incidence, and Voigtländer and Voth regarded the Biraben data as plague epidemics. These 3 terms are not interchangeable. The lack of clarity on what the data set represents has led to the drawing of false conclusions.

Which of the 3 graphs uses the correct terminology? In fact, none of them do. The data collected by Biraben represent the availability of sources mentioning plague and not the severity or pervasiveness of the disease in any given year. More narrowly defined, the data set represents those sources Biraben was able to find in the timespan of researching his book while working in Paris. In no way does this data set represent the full coverage of all historical plague activity throughout the whole of Europe.

Furthermore, the Biraben data set has an urban bias. Most of the mentions of plague occurrences (particularly those outside of France) pertained to cities, perhaps because urban documents were more easily accessible. For example, in the database used to create Figure 1, the city of Paris was indicated as having 90 plague outbreaks, yet the middle-sized town of Soissons ≈100 km to the northeast only experienced 3 during the same period (1347–1760). We must view this result skeptically, given that this number would have meant that a new plague outbreak in Paris occurred on average every 3.5 years over a period of 320 years (the last plague in northern France was in the late 1660s). This average rate contradicts a wealth of scholarship that suggests that, after the Black Death, the average interval separating 2 plague occurrences in Northwest Europe was around 11–12 years in the 14th century, decreasing to 15–20 years by the late 15th century (*22*), and being anything from 10 to 20 years by the 17th century (*18*,*23*). Ultimately, by confusing mentions of plague in available sources as a representation of individual incidences or outbreaks of the disease, Biraben’s data set has led to a gross overestimation of plague in big cities and a gross underestimation of plague in smaller towns and villages. This confusion is problematic, considering some scholars have linked plague spread to commerce (*9*), trade routes (*7*), or distance to navigable rivers (*6*), all factors highly conducive to the development of cities (*24*). Misinterpretation of Biraben’s data set also feeds into a narrative describing plague as a fundamentally urban phenomenon when research is beginning to reveal this perception to be a fallacy (*14*,*19*).

Next, we address the question of how the data were collected. The collection process did not aim to attain a representative sample of all historical plague outbreaks across Europe, which would have been necessary for a data set attempting to offer a long-term pan-European overview. As previously mentioned, the data set has crucial gaps in geographic coverage; it does not provide an unbiased sample for every region in Europe and, within many regions, provides a clear urban bias. However, substantial gaps are evident in temporal coverage as well. For instance, the original data set gives the impression that the 16th and 17th centuries witnessed much higher plague activity than the 14th and 15th centuries. Yue et al. are especially not critical in this regard and suggest that more severe plague outbreaks occurred during the Thirty Years’ War (1618‒1648) than other periods (*6*), an association also suggested in other studies (*11*). Despite the fact that long and devastating wars occurred similarly throughout the 14th through 16th centuries in many parts of Western Europe, no consideration is given to why the Thirty Years’ War would set off more severe plagues than, for example, the Hundred Years’ War (1337‒1453). Furthermore, absence of evidence cannot be interpreted as evidence of absence in the case of late-medieval plague outbreaks (*25*). The literature has explicitly pointed out the paucity of quantifiable evidence for the recurring epidemics of the late Middle Ages (1349–1500) (*26*); however, this paucity is also related to a polarization of the research focus between the initial Black Death outbreak and early modern outbreaks. To interpret both the incomplete recording of sources by Biraben and the less forthcoming nature of late-medieval plague documents as evidence of lower plague activity is unsatisfactory. This interpretation accepted by some researchers is yet another reason why the noncritical use of the data leads us to consternation over the results and interpretations produced.

Last, we address the third and final problem, the absence of source critique. Despite elucidating the basic symptoms one would expect to see with bubonic plague, such as fever, buboes, and vomiting, Biraben never justified how he came to identify certain localities in certain years as experiencing plagues in his own data set, and a structural overview of the original sources he used is missing. Because of his inadequate citation practices, we have little hope of checking the validity of Biraben’s assertions, which undermines the reliability and accuracy of the data set that has been reused on a number of occasions by others. A further problem with not knowing the original sources is that equal weight in terms of accuracy and reliability cannot necessarily be attributed to different reference types (e.g., resources allowing for quantification of mortality rates, administrative sources with qualitative direct mentions of plague, and narrative sources with qualitative direct mentions of plague) (*17*). This problem is magnified further in light of increasing interest in germ theory‒based nosology and the retrospective diagnosis of diseases (*1*). Medieval historians question the methods used for identifying diseases in the past (*27*,*28*). Laboratories have confirmed *Yersinia pestis* in burial sites connected to the initial Black Death outbreak of 1347–1352 (*29*), but few works have explicitly linked *Y. pestis* to burial sites of other specific, recurring, late-medieval plague outbreaks (*25*).

Accordingly for other late-medieval plagues, we are often reliant on anecdotal references by contemporaries in the absence of laboratory or even epidemiologic evidence. Using references by contemporaries is problematic, given the terms *peste* or *pestilentia* were often indiscriminate references to all sorts of afflictions (*30*). Only starting roughly around the second half of the 15th century do we find more explicit differentiation in the descriptions of diseases in the Low Countries and Italy (*31*,*32*), and even these descriptions still were by no means systematic. For many of the putative late-medieval outbreaks after the initial Black Death, most literary sources do not mention key signs or symptoms, such as the combination of buboes, fever, and a rapidity of death. When signs or symptoms are referenced, they are fragmentary and localized and, therefore, difficult to use as evidence for the occurrence of general epidemic outbreaks over large territories. Even in the early modern period, when disease differentiation became much more commonplace in sources (*19*,*33*), not every death spike could be accounted for, especially during periods such as the Thirty Years’ War when a host of other diseases were present alongside plague (*18*). We must also bear in mind that even in modern times the diagnosis of plague on the basis of signs and symptoms is problematic for trained medical professionals (*34*). The only way to determine the etiologic agent responsible for a disease is by using molecular diagnostic tests. In other words, we are not sure that the mentions of plague identified originally by Biraben were in fact plague at all, especially for the medieval period, and as mentioned, we have no way of checking Biraben’s data set without citations to the original manuscripts. This problem is further illuminated by some of the plagues Biraben identified, such as the plague of 1437–1440, which occurred during a period of extreme cold weather (*35*,*36*) and manifested as harvest failures and famine-related diseases (*37*); research has suggested that waterborne infections were more likely the cause of this pestilence (*38*).

## Moving Forward

**Figure 7:**
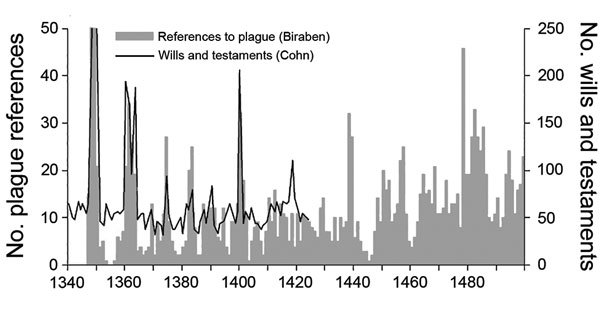
Comparison of Biraben’s and Cohn’s historical plague data sets. Biraben’s data set included references to plague in various types of documents from Italy, Iberia, France, the Low Countries, and the British Isles that were written during 1345–1499 (*2**,**3*). Cohn’s data set included information from a select set of documents (wills and testaments of 9 cities) that were drafted during 1340–1424 (*21*). Graph provided courtesy of Campbell B. The great transition: climate, disease and society in the late-medieval world. Cambridge: Cambridge University Press; 2016 (*39*).

We suggest 3 necessary steps to take to rectify some of the mistakes made with the use of digital databases of plague, which were often constructed by using Biraben’s data. First, if we are going to pursue the Biraben database, we at least need to check his plague references with other forms of evidence rather than taking him at his word. Historians have done this in previous years with care by using only a select geographic sample of Biraben’s evidence and comparing the data to other quantifiable indexes, such as the temporal distribution of will production (Figure 7) (*39*).

Second, scholars looking to test certain hypotheses, such as the effect of navigable rivers, commercialization, trade routes, or climatic fluctuations, should do so by using a historical plague data set of a much more restricted geographic or temporal scope to limit problems such as the inequalities in availability of source material or scholarly attention. We need to escape the confines of excessively localized and excessively macro scales and, instead, reap the benefits of a more workable historical laboratory at a regional level (*40*). This restriction method is similar to how epidemiologists try to control for confounders by limiting their data to a specific group of persons sharing a specific characteristic. A way of implementing this in practice is by moving away from using data sets that consolidate different kinds of references to plague through different kinds of evidence (often without justification) and moving toward using data sets that can show differences in plague characteristics by comparing the same type of source material, a method that offers greater control. For example, by using only data from church burial records from the 16th and 17th centuries over many parts of Europe, a systemic comparison can be performed between urban and rural localities over time and with regard to plague severity, seasonality, pervasiveness, and various kinds of selectivity (*14*,*18*). Epidemiologic information on plagues is better provided by using this approach than by using a random set of diverse manuscripts that may or may not refer to plague.

Third, it is clear that new databases of plague incidence have to be compiled by historians using data sets besides Biraben’s. Given that this task is laborious and time-consuming, incentives are needed for historians to compile this information. One incentive could be the formal inclusion of trained medieval historians in large interdisciplinary scientific teams interested in charting and explaining the spread of plague.
